# Inverse Association between Omega-3 Index and Severity of COVID-19: A Case–Control Study

**DOI:** 10.3390/ijerph19116445

**Published:** 2022-05-25

**Authors:** Muriel Ramírez-Santana, Rodrigo Zapata Barra, Marcela Ñunque González, José Miguel Müller, Juan Enrique Vásquez, Franco Ravera, Gustavo Lago, Eduardo Cañón, Daniella Castañeda, Madelaine Pradenas

**Affiliations:** 1 Public Health Department, Faculty of Medicine, Universidad Católica del Norte, Coquimbo 1780000, Chile; 2 Faculty of Medical Science, Universidad de Santiago de Chile and Neurosurgery Service, Hospital Regional Libertador Bernardo O’Higgins, Rancagua 2820000, Chile; rzapata.barra@gmail.com (R.Z.B.); jmmullerr@gmail.com (J.M.M.); kikevas@hotmail.com (J.E.V.); fraveraz@gmail.com (F.R.); 3 Clinical Department, Faculty of Medicine, Universidad Católica del Norte, Coquimbo 1780000, Chile; marcela.nunque@ucn.cl; 4 Hospital Clínico Fusat, Rancagua 2820000, Chile; drlagogus@gmail.com; 5 Hospital Regional Libertador Bernardo O’Higgins, Rancagua 2820000, Chile; eduardo.canonaedo@gmail.com (E.C.); elsadaniella@gmail.com (D.C.); mpradenasramirez@gmail.com (M.P.)

**Keywords:** omega-3 fatty acids, COVID-19, Omega-3 Index, inflammation

## Abstract

Background: Omega-3 fatty acids enhance immune response and modulate inflammation. This study aimed to determine the relationship between omega-3 fatty acid status and the severity of SARS-CoV-2 infection. Methods: Using a case–control design, we compared hospitalized patients with severe SARS-CoV-2 infection (cases, *n* = 73) with a group of ambulatory patients with mild SARS-CoV-2 infection (controls, *n* = 71). No patients were vaccinated against SARS-CoV-2. Results: The cases were older (*p* = 0.003), less educated (*p* = 0.001), had larger neck and smaller waist circumferences (*p* = 0.035 and *p* = 0.003, respectively), more frequently had diabetes (*p* < 0.001), consumed less fish (*p* < 0.001), consumed higher proportions of fried fish (*p* = 0.001), and had lower Omega-3 Index (O3I) values (*p* = 0.001) than controls. Cases had significantly lower rates of upper airway symptoms than controls. Lower O3I was associated with an increased likelihood of developing severe COVID-19 after adjusting for potential confounders (OR: 0.52; CI 0.32–0.86). Diabetes (OR: 4.41; CI 1.60–12.12), neck circumference (OR: 1.12; CI 1.03–1.21), and older age (OR: 1.03; CI 1.002–1.062) were also linked to COVID-19 severity. Fried fish consumption and low educational level were independent risk factors for severe COVID-19. Conclusions: This study suggests incorporating nutritional interventions to improve omega-3 status within nonpharmacological measures may help to reduce the severity of COVID-19.

## 1. Introduction

Immunization against coronavirus disease 2019 (COVID-19) has been demonstrated to effectively reduce the severity of disease [1,2]. However, the vulnerability of high-risk individuals to COVID-19 and the low vaccination rates achieved in developing countries still remain serious issues of public health concern [3,4]. Although airway mucosal immunity may play a crucial role in preventing progression to the more severe spectrum of COVID-19, easy-to-implement interventions to enhance airway mucosal immunity have not yet been identified. Growing evidence supports the hypothesis that both homeostasis of inflammation resolution and airway mucosal immunity may be largely mediated by a novel superfamily of autacoids, now termed specialized proresolving mediators (SPMs); the majority of SPMs are biosynthesized from the long-chain omega-3 fatty acids eicosapentaenoic acid (EPA) and docosahexaenoic acid (DHA) [5,6,7,8,9]. Importantly, a deficiency of EPA and DHA has been detected in populations throughout the world, and this issue is particularly severe in Western populations [10]. Blood levels of EPA and DHA can be reliably determined by the Omega-3 Index (O3I), which is the content of EPA and DHA expressed as a percentage of the total fatty acid content of RBC membranes. Long-term intake of omega-3 fatty acids is the main predictor of the O3I, though other factors are also involved [11,12,13]. Numerous health benefits have been described for omega-3 fatty acids, including the ability to enhance the immune response and modulate inflammation [14]. Blood levels of omega-3 fatty acids have been inversely associated with the risk of premature death in adults [15]. Furthermore, inverse associations between the O3I and mechanical ventilation and the risk of death were recently reported in a cohort of patients with severe COVID-19 [16]. Here, we report the findings of a case–control study comparing the O3I in patients with mild and severe COVID-19.

## 2. Materials and Methods

### 2.1. Sample Size and Selection of Participants

This case–control study was conducted in two cities in Chile. The selection of the cities was based on convenience, according to the residence, place of work, and specialty of the researchers. The sample size required for the case–control study was calculated based on a frequency of exposure of 90% for cases and 70% for controls. The minimum number of cases and controls (equal numbers per group) required to detect an OR of 1.5, with 80% statistical power and 95% significance, was 62 individuals per group. The group of cases (*n* = 73) were enrolled in Rancagua city between November 2020 and April 2021. The controls (*n* = 71) were enrolled in La Serena city between June and December 2021. All patients were adults, with a confirmatory diagnosis of COVID-19 based on a reverse-transcriptase polymerase chain reaction (PCR) test taken with a nasal swab, did not have a previous history of COVID-19 vaccination, and were enrolled after providing their informed consent to participate. Cases were patients hospitalized with respiratory failure due to COVID-19 at Rancagua Regional Hospital. These patients were evaluated by the treating medical team and a trained nutritionist collected their blood samples. The control group was patients who presented mild SARS-CoV-2 infection. During recruitment, each case was intended to be matched to a control of similar age and sex. These patients were evaluated by trained nutrition students at primary health centers in La Serena city, under the supervision of a senior nutritionist.

### 2.2. Variables and Sources of Information

The dependent variable was the clinical status of COVID-19 (mild or severe). The independent variables were sociodemographic features (sex, age, educational level, health insurance), history of comorbidities, body composition (BMI, waist circumference, neck circumference, arm circumference, bicipital and triceps fold), Omega-3 Index (O3I), and consumption of fish and supplements. Symptoms were also recorded. Direct measurements of biometric indices and blood samples were obtained for the patients with severe COVID-19 during their in-hospital stay; a structured in-person interview was also conducted with the patient or a close relative.

### 2.3. Laboratory Analysis and Quality Control

To determine the Omega-3 Index, a drop of blood was placed on a dry blood spot (DBS) collection card provided by the OmegaQuant^®^ laboratory (Sioux Falls, SD, USA) and kept frozen at −2 °C before shipment to the laboratory in the USA, following the laboratory’s instructions. Additionally, blood samples were collected at different time points throughout the study period from 18 healthy subjects as quality controls for the process of storing and shipping the DBS collection cards. Most of these quality control subjects had been taking omega-3 fatty acid supplements (dose range of EPA + DHA between 1 and 2 g) daily for at least 4 months. The laboratory remained blinded to the grouping of the subjects. Details of the cases and laboratory protocol were published [16].

### 2.4. Data Registration and Analysis

Information was collected using an online form that generates an Excel spreadsheet. The data analysis was performed in SPSS (V26) IBM Statistics^®^ (Armonk, New York, NY, USA). Standard descriptive analysis (means and standard deviations, counts, and percentages) was used to assess the data. The characteristics of the controls and cases were compared using the Mann–Whitney *U*-test or Pearson’s Chi-squared test, depending on the nature of the variables. Finally, a logistic regression model was executed to assess if the O3I was related to clinical status (mild or severe), with adjustment for potential confounders (age, diabetes, neck circumference, waist circumference). For all tests, statistical significance was defined as *p* < 0.05 (two-sided).

### 2.5. Ethical Approval

The study protocol was approved by the Ethics Committee of the Faculty of Medicine of Universidad Católica del Norte (Resolution Number: 26/2021) and the Ethics Committee of the Regional Health Service (Resolution: 13 October 2021).

## 3. Results

The characteristics of the case and control groups are presented in Table 1. There were no differences in the sex distribution, health insurance, or tobacco consumption between the groups, though the cases had a higher average age (*p* = 0.003) and lower educational level (*p* = 0.001) compared to the control group. The cases also had a significantly higher frequency of diabetes (*p* < 0.001) and were more likely to use corticosteroids prior to infection (*p* = 0.08), although the latter difference was not significant. The distribution of other comorbidities was not significantly different between the study groups. In terms of body composition, the cases had a larger neck circumference and smaller waist circumference than the controls (*p* = 0.035 and *p* = 0.003, respectively).

There were striking differences in fish consumption and also the method of cooking fish between cases and controls: the cases consumed less fish, and when they did, more frequently consumed fried fish (*p* < 0.001). In contrast, the controls not only consumed more fish, but also more frequently consumed raw, canned, or cooked fish without frying (*p* = 0.001). There were no differences in the types of fish consumed between groups; in general, the types of fish consumed contain low proportions of omega-3 fatty acids [17]. Finally, the controls had significantly higher O3I values than cases (*p* = 0.001). The cases had an average O3I of 4.15% (max. 6.14% and min. 3.06%) and the controls 4.57% (max. 7.26% and min. 2.54%). The quality control individuals (*n* = 18) had a mean O3I of 7.58%, with a maximum of 10.71% and minimum of 4.65%.

Table 2 compares the frequency of symptoms between the study groups. Significantly higher proportions of cases had respiratory distress (*p* > 0.001), fatigue or prostration (*p* = 0.002), and cough (*p* = 0.003) than controls. The frequency of upper airway symptoms was significantly lower among cases than controls. There were no differences in the frequency of fever and chest pain between the two groups.

Analysis of the variables associated with clinical outcome (severe or mild COVID-19) is presented in Table 3. We included the variables that were significantly different between the groups in this analysis. Of these variables, only waist circumference was found to have no influence on clinical outcome (OR: 0.999; CI 0.994–1.004). The variables with the greatest influence on clinical outcome were the presence of diabetes (OR: 5.37; CI 2.15–13.38), low educational level (OR: 5.24; CI 2.54–6.99), and the consumption of nonfried fish (OR: 0.208; CI 0.09–0.482), with the latter being a significant protective factor. The O3I was also associated with the severity of SARS-CoV-2 infection (OR: 0.475; CI 0.3–0.725), as well as age (OR: 1.038; CI 1.011–1.065) and neck circumference (OR: 1.107; CI 1.024–1.197).

The variables that were significant in the individual analysis were incorporated in a logistic regression model for the association between the O3I and the severity of COVID-19. Two interactions were detected: firstly, between the consumption of nonfried fish and the O3I and, secondly, between age and education. Thus, to evaluate the influence of the O3I on clinical status, the model was adjusted for the significant (and biological) variables showing no interaction. High levels of omega-3 fatty acids in blood (i.e., a higher O3I) were associated with a 48% reduction in the risk of serious illness (from between 68% and 14%, approximately). Diabetes remained the second most important risk factor for severe disease, followed by neck circumference and older age.

## 4. Discussion

In this case–control study, we observed an inverse association between the O3I and the severity of COVID-19, even after adjusting for confounding factors. In agreement with our previous publication, we found the O3I inversely correlated with both the risk of hospitalization and major clinical endpoints of severe COVID-19 [16]. Importantly, the O3I values obtained in our study reliably reflect the omega-3 status of the subjects, as highlighted by the substantial difference detected in the O3I values of the patients and the quality control subjects, most of whom regularly consume omega-3 supplements. These findings are in agreement with three other studies that were included with our previous work in a recent review [18]

The consistent inverse correlation between O3I and clinical status within a relatively narrow range of O3I values strongly suggests omega-3 status may play a crucial role in the severity of COVID-19. However, the results of recent randomized controlled trials (RCTs) of omega-3 fatty acids in COVID-19 are controversial. None of the RCTs that investigated the effects of omega-3 fatty acid supplementation measured the Omega-3 Index, and only one of these RCTs included DHA as an integral part of the intervention [19,20,21]. The internal validity of these RCTs is questionable because the failure to include the O3I as a reliable biomarker of the omega-3 status of patients does not enable valid comparisons to be made between the RCT arms; clinical events correlate more significantly with blood levels of EPA and DHA than with the supplemented amounts of these fatty acids. Additionally, the incorporation profile of orally administered omega-3 fatty acids in functional and structural lipid pools is limited in the short-term. The lack of DHA as an integral part of the interventions in the VASCEPA-COVID-19 and PREPARE-IT 1 and 2 RCTs is also a serious flaw in the design of these studies. DHA is the most abundant omega-3 fatty acid in membrane phospholipids and is present in all organs [20,21]. DHA is also the precursor of the majority of omega-3 fatty-acid-derived specialized proresolving mediators (SPMs), i.e., the D-series resolvins, maresins, and protectins [9]. These lipid mediators attenuate viral replication and also promote the resolution of inflammation [5,22,23]. In experimental models, protectin D1 exhibited potent antiviral bioactivity against the virulent H1N1 influenza virus strain [22]. Interestingly, we also detected statistically significant differences in DHA levels in RBCs between cases and controls (data not shown).

As has been shown in other studies, older persons and people with comorbidities are at higher risk of becoming seriously ill and dying of COVID-19 [24,25]. In our study, the severe cases were significantly older and had a higher frequency of diabetes, and diabetes was significantly associated with the severity of COVID-19 in our regression model. Prognostic indicators of metabolic dysfunction such as type 2 diabetes and central adiposity (as indicated by neck circumference measurements) were significantly higher in cases than controls. Importantly, metabolic dysfunction and unhealthy dietary habits (such as frying as a means of cooking) are hallmarks of Western-diet-induced obesity, and a proinflammatory connection between the Western diet and the immune system has recently been recognized [26]. Although several pathogenetic mechanisms may be involved in this inflammatory connection, a potentially important interference related to the metabolism of omega-3 fatty acids has largely been overlooked. Generation of SPMs may not only depend on adequate omega-3 fatty acid tissue levels, but also on the efficiency of the related biosynthetic routes [9]. Evidence from experimental and human studies suggests the Western diet and obesity enhance the production of proinflammatory mediators and impair the SPM signature, which could lead to an adverse outcome in COVID-19 [27].

On the other hand, SPM receptors have been identified on human airway epithelial cells, as well as other cells directly related to inflammation at mucosal epithelial borders (such as neutrophils, eosinophils, mast cells, monocytes, macrophages, lymphocytes, dendritic cells, innate lymphoid cells, and natural killer cells) [9]. Interestingly, increased serum levels of SPMs have recently been identified in patients with severe COVID-19, and the levels of SPMs correlated with markers of the adaptive immune response [28]. The wide distribution of SPM receptors among cells of both the innate immune system and structural cells of the airway strongly suggests SPMs may play a crucial role in orchestrating a coordinated immune response in airway diseases [28]. The variability in the function of the innate immune system among individuals has recently been regarded as a major determinant of the heterogeneity of the severity of COVID-19. Thus, a coordinated local and systemic immune response may be crucial to prevent the more severe spectrum of COVID-19 [29,30]. Patients with severe COVID-19 may not be capable of mounting an effective mucosal immune response in the upper airway. Interestingly, the rates of upper airway symptoms were significantly lower among cases than control subjects in this study, consistent with reports by the CDC [31,32]. This strongly suggests that the patients with severe disease were not able to mount an effective immune response at the mucosal level of the upper respiratory tract. It is worth mentioning that patient recruitment occurred during the first wave of the COVID-19 pandemic, when patients were naturally infected with the original virus, and during the second wave, when the Gamma and Delta variants predominated [33]. Unfortunately, the omicron variant, which is associated with higher frequencies of upper respiratory symptoms, was not circulating at the time of recruitment [32,33].

Obesity has also been reported as an important risk factor for severe COVID-19 [34,35]. There is consensus that waist circumference represents an indicator of the distribution of body fat at the intra-abdominal level. Given its high proinflammatory activity, visceral fat deposits increase the risk of metabolic syndrome and type 2 diabetes and could contribute to a poorer inflammatory prognosis in patients with larger waist circumferences [36]. However, the reliability of this indicator is doubtful, as waist circumference may be affected by abdominal distension and differences in the measurement technique between hospitalized or resting patients. Additionally, other pathologies that cause abdominal distension may also be present. The average waist circumference of both cases and controls exceeded the cut-off points for central obesity in men (≤90 cm) and women (≤80 cm) defined by the World Health Organization [37]. Thus, one possible explanation for the fact that waist circumference did not emerge as a significant factor for clinical outcome could lie in the fact that both groups presented comparably high measurements, as well as similarly high body mass indexes (BMIs).

On other hand, the cases had significantly higher neck circumferences than the controls. This indicator has the advantage of being easy to measure, does not change during the day, is not influenced by abdominal distension caused by ingested food, and is not altered by inhalation or exhalation. A systematic review indicated that neck circumference is directly associated with the percentage of total fat mass, measured via bioimpedance, the most accurate technique for measuring total body fat [38]. Chronic inflammation associated with obesity and its multiple systemic repercussions are characterized by increased production of proinflammatory adipocytokines [39], persistent activation of inflammatory pathways, and a deficit of mediators specialized in resolution. Combined with the inflammatory cascade caused by COVID-19, these factors could lead to a poorer prognosis in patients with larger neck circumferences. A study that performed standard tests of pulmonary function and static respiratory muscle strength found that individuals with neck circumferences greater than or equal to 43 cm had less resistance in the inspiratory muscles, while abdominal adiposity did not affect respiratory muscle strength [40]. Thus, this evidence may explain the relationship between neck circumference and clinical outcome. Moreover, we suggest that neck circumference may represent a simple, easily accessible indicator of the prognosis of patients with COVID-19 that is more reliable than BMI or waist circumference.

A relationship between socioeconomic factors and SARS-CoV-2 infection has been previously reported in Chile [41,42]. Moreover, studies show that the quality of diets worldwide has reduced due to the pandemic, and these changes are associated with an increased risk of severe infections [43]. The difference in the level of education between severe cases and controls (mild infection) is not surprising in the context of Chile, where education is directly associated with economic status and access to quality food [44]. Despite being a coastal country, access to fish is limited by economic factors. According to the national nationwide health survey, only 9.2% of the population consumes fish two or more times a week [45,46]. Additionally, the method of cooking fish matters. The cases not only consumed less fish than the controls, but also tended to eat fried fish, which reduces the nutritional characteristics of omega-3 fatty acids [47,48]. Furthermore, eating unfried fish was identified as another variable of interest in the relationship between the O3I and the severity of infection. In our preliminary study of severe cases, we also observed higher consumption of fried fish among patients with lower O3I levels [16].

One important strength of this study is the evaluation of patients without a history of vaccination; thus, we can exclude the influence of vaccines on the risk of serious symptoms. Moreover, the assessment of quality controls for the O3I made it possible to assess the technique and eliminate possible interference in the storage and shipment of samples outside the country for analysis.

In terms of the limitations of this study, recruitment of subjects from different populations during different waves of COVID-19 is the main limitation of our study. However, infection with the Delta variant has been associated with increased likelihood of hospitalization or experiencing more severe disease, which should have increased the likelihood of more severe disease in the control group [49]. On other hand, the cases and controls are residents of different cities. It may be possible that people in these cities have different eating habits and access to fish. However, while this may contribute to the origin of different levels of omega-3 in the blood, it does not interfere with the relationship between the O3I and clinical status. Regarding fish consumption, we considered the frequency of consumption, type of fish, and cooking technique, but not the amounts of fish or omega-3 EPA-DHA consumed. Further study of these parameters could help to more precisely determine the recommended quantity of fish that should be consumed to obtain beneficial effects among the general population. Finally, other nutritional variables that have been related to the immune response were not measured, such as the levels of vitamins and other microelements.

The results of this study are of great interest in relation to modifiable factors that may help to prevent serious SARS-CoV-2 infection in a pandemic situation. The consumption of fish or omega-3 supplements may significantly reduce the social and healthcare costs of severe COVID-19, by decreasing the proportion of patients who develop severe infections. Diet has previously been indicated as a modifiable factor related to COVID-19, and similar measures have already been suggested by other investigators [14,50,51]. Consequently, public health interventions may consist of promoting adequate fish consumption among the general population, in terms of both quantity (at least three times per week) and quality (type of fish and method of cooking). Additionally, offering supplements through primary healthcare could be suggested, focusing on specific groups at risk of severe COVID-19, such as healthcare personnel and patients with chronic diseases. Finally, social determinants such as education and access to quality food remain factors of interest that need to be addressed.

## 5. Conclusions

The level of omega-3 fatty acids is a nutritional factor of high interest in terms of the clinical outcomes of SARS-CoV-2 infection, as a low Omega-3 Index was associated with an increased likelihood of developing severe COVID-19, independently of other variables. The study results suggest that other factors such as older age, low educational level, presence of diabetes, and a large neck circumference (as a measurement of obesity) may be linked to the severity of COVID-19. Both the frequency of consumption of fish and the method of cooking fish were also important factors, and we recommended that fish should not be fried. Overall, the results of this study suggest incorporation of nutritional interventions within nonpharmacological measures may help to reduce the severity of COVID-19.

## Figures and Tables

**Table 1:** Characteristics of case and control groups.

Variable		Cases		Controls		^a^*p*-Value
		Number	Percentage	Number	Percentage	
Sex	Male	38	52.1	35	49.3	0.868
	Female	35	47.9	36	50.7	
Health insurance	FONASA	72	98.6	67	94.4	0.352
	ISAPRE	1	1.4	3	4.2	
	Other	0	0.0	1	1.4	
Education	Primary education or never attended	35	47.9	17	23.9	0.001
	Secondary education	31	42.5	31	43.7	
	Technician or professional	7	9.6	23	32.4	
Comorbidity	Cancer	0	0.0	1	1.4	0.21
	Diabetes	27	37.0	7	9.9	<0.001
	Hypertension	34	46.6	21	29.6	0.41
	Cardiomyopathy	5	6.8	0	0.0	0.03
	HIV-AIDS	1	1.4	0	0.0	0.37
	Chronic respiratory disease	12	16.4	7	9.9	0.31
	Chronic kidney disease	5	6.8	1	1.4	0.16
	Corticoids use	2	2.7	1	1.4	0.08
Tobacco consumption		6	8.2	11	15.5	0.2
Omega-3 supplementation		3	4.1	5	7.0	0.49
Frequency of nonfried fish consumption						
	One or more times a week	31	42.5	33	46.5	<0.001
	One or two times a month	12	16.4	29	40.8	
	No consumption (or always fried)	30	41.1	9	12.7	
Method of cooking fish						
	Always fried	19	26.0	5	7.0	0.001
	Oven, griddle, pot, canned, raw, or fried	10	13.7	9	12.7	
	Oven, griddle, pot, canned, raw	33	45.2	53	74.6	
	No consumption	11	15.1	4	5.6	
Type of fish	Over 300 mg O3/100 g (salmon, mackerel, sawfish)	14	19.2	13	18.3	0.163
	200–300 mg O3/100 g (tuna, hake, croaker, pippin)	48	65.8	53	74.6	
	Does not consume	11	15.1	4	5.6	
		Cases		Controls		^b^*p*-value
		Mean	S.D.	Mean	S.D.	
Age		59.37	13.43	52.76	13.49	0.003
Body composition						
	BMI	29.53	6.21	29.22	4.2	0.846
	Neck circumference (cm)	40.57	5.13	38.49	3.89	0.035
	Waist circumference (cm)	106.26	14.33	110.03	95.09	0.003
	Arm circumference (cm)	32.67	4.12	32.18	3.04	0.552
	Triceps fold (mm)	19.93	8.21	19.65	6.94	0.938
	Bicipital fold (mm)	16.14	8.71	13.76	6.81	0.102
Fatty acids in blood						
	Omega-3 Index (%)	4.147	0.693	4.574	0.818	0.001

^a^: Pearson’s Chi-squared test; ^b^: Mann–Whitney *U*-test.

**Table 2:** Comparison of the frequency of symptoms between the study groups.

Symptom	Cases		Controls		*p*-Value *
	Number	Percentage	Number	Percentage	
Respiratory distress	69	94.5	18	25.4	<0.001
Fatigue/prostration	43	58.9	24	33.8	0.002
Cough	37	50.7	19	26.8	0.003
Fever	31	42.5	21	29.6	0.075
Myalgia	23	31.5	44	62.0	<0.001
Headache	8	11	34	47.9	<0.001
Odynophagia	5	6.8	17	23.9	0.004
Chest pain	4	5.5	12	16.9	0.052
Loss of smell	3	4.1	29	40.8	<0.001
Loss of taste	3	4.1	28	39.4	<0.001
Abdominal pain/diarrhea	2	2.7	22	31.0	<0.001

* Pearson’s Chi-squared test.

**Table 3:** Raw ORs and logistic regression model of variables influencing the clinical outcome of SARS-CoV-2 infection.

Variable	Raw OR	CI	*p*-Value	Logistic Regression Model		
				Adjusted OR	CI	*p*-Value
Omega-3 Index *	0.475	0.3–0.725	0.001	0.521	0.316–0.86	0.011
Age *	1.038	1.011–1.065	0.005	1.031	1.002–1.062	0.039
Diabetes (yes)	5.366	2.152–13.38	<0.001	4.405	1.601–12.12	0.004
Neck circumference *	1.107	1.024–1.197	0.011	1.115	1.025–1.213	0.011
Waist circumference *	0.999	0.994–1.004	0.741			
No fried fish consumption	0.208	0.09–0.482	<0.001			
*Education*						
Above secondary education (ref)			0.007			
Secondary education	2.536	0.919–6.998	0.072			
Primary education	5.24	1.83–15.01	0.002			

CI: confidence interval; OR: odds ratio; * continuous variable.

## Data Availability

Data can be requested from the corresponding author.
